# Optimizing bioavailability of oral administration of small peptides through pharmacokinetic and pharmacodynamic parameters: The effect of water and timing of meal intake on oral delivery of Salmon Calcitonin

**DOI:** 10.1186/1472-6904-8-5

**Published:** 2008-09-09

**Authors:** Morten A Karsdal, Inger Byrjalsen, Bente J Riis, Claus Christiansen

**Affiliations:** 1 Nordic Bioscience A/S, CCBR, Herlev/Ballerup, DK-2730 Herlev, Denmark

## Abstract

**Background:**

To investigate the influence of water intake and dose timing on the pharmacokinetic and pharmacodynamic parameters of an oral formulation of salmon calcitonin (sCT).

**Methods:**

The study was a randomized, partially-blind, placebo-controlled, single dose, exploratory crossover phase I study. 56 healthy postmenopausal women were randomly assigned to receive five treatments. The treatments comprised a combination of study medication (SMC021 (0.8 mg sCT + 200 mg 5-CNAC), SMC021 placebo, or 200 IU Miacalcic^® ^NS nasal spray), water volume given with the tablet (50 or 200 ml water), and time between dosing and meal (10, 30, or 60 minutes pre-meal). Plasma sCT levels and changes in the bone resorption (C-terminal telopeptide of collagen type I) was investigated. Trial regristration

**Results:**

Oral delivery of 0.8 mg of sCT with 50 ml of water compared to that with 200 ml water resulted in a two-fold increase in maximum concentration (C_max _and AUC_0–4_) of plasma sCT but comparable time to reach maximum concentration (T_max_). The sCT AUC_0–4 _with 50 ml of water was 4-fold higher than that obtained with nasal calcitonin. The increased absorption of sCT resulted in increased efficacy demonstrated by AUC of the relative change of serum CTX-I measured in the 6 hours post dosing.

**Conclusion:**

0.8 mg sCT with 50 ml of water taken 30 and 60 minutes prior to meal time resulted in optimal pharmacodynamic and pharmacokinetic parameters. The data suggest that this novel oral formulation may have improved absorption and reduction of bone resorption compared to that of the nasal form.

## Background

Calcitonin is a natural peptide hormone produced by parafollicular cells (C-cells) in the thyroid gland [1,2], which is secreted in response to excess calcium in the serum [3]. Calcitonin was discovered more than 40 years ago [4,5], and possesses potent anti-resorptive effects. Calcitonin binds directly to the calcitonin receptor on osteoclasts which result in a transient reversible inhibition of bone resorption [6].

Calcitonin (CT) is approved for the treatment of osteoporosis and other diseases involving accelerated bone turnover [6,7]. Calcitonin treatment has until now been limited to either subcutaneous or intranasal administration, that occasionally is hampered by administration difficulties and local irritation [6]. Some subjects are reluctant to administer drugs intranasal or parenteral limiting the potential treatment population. An oral form of calcitonin may improve subject acceptance and facilitate improved subject compliance by providing a more acceptable, alternative delivery route for peptide therapy [6]. Thus, an increasing number of investigations have focused on formulating calcitonin to provide a novel oral formulation that includes but not are limited to [6-18].

Oral drug delivery of small peptides is hampered by an array of obstacles [19,20], including but not limited to degradation by proteolytic enzymes in the digestive tract and secondary intestinal uptake [19-21]. These difficulties are best illustrated by the complete lack of peptides in oral formulations approved by the FDA.

Due to the short half-life of calcitonin in serum several attempts have been applied to increase plasma concentrations. One very recent approach of oral calcitonin formulation is the use of the Eligen technology [6]. This carrier [7,22] interacts with the drug molecule to create an insoluble entity at low pH which later dissolves and facilitates intestinal uptake, by enhancing peptide transport over the non-polar biological membrane [22]. This has been demonstrated to result in clinical efficacy [6,7].

Even in the face of already documented clinical efficacy with this formulation [6,7], plasma concentrations of sCT may be further optimized through pushing of gastric emptying that would result in rapid transit of calcitonin through the gastrointestinal tract [23]. This may partly circumvent calcitonin degradation by the vast array of endopeptidases and other enzymes in the gastric tract optimally functioning at low pH [19,20,23]. Several techniques have been applied which prolong or shorten the retention in the gastric tract, with more or less success [19,20,24,25], in which the effect of water remains to be carefully investigated in particular with special emphasis on peptide uptake.

The primary objective of the current study was to investigate the effects of two volumes of water used to take a single SMC021 tablet and the effect of three different post-dose meal timings on the pharmacokinetic (PK) and pharmacodynamic (PD) profile of 0.8 mg sCT, as assessed by C_max _and AUC_0–4 _of sCT and bone resorption by CTX-I. Additionally the PK and PD profiles of intranasally administered sCT (Miacalcic^® ^200 IU NS) were evaluated for comparison in a small number of subjects; this treatment was not blinded and therefore comparisons may be affected by blinding bias.

## Methods

### Drug substance

SMC021 is an oral formulation of salmon calcitonin. It consists of the peptide hormone and 5-CNAC, {8-(N-2-hydroxy-5-chloro-benzoyl)-amino-caprylic acid)} a unimolecular enhancer of gastrointestinal peptide absorption, developed by Emisphere Technology, Inc. and licensed to Novartis. The moiety was designed to deliver peptides orally without significant enzymatic hydrolysis or alteration of the biological characteristics of the peptide. When 5-CNAC is administered orally in combination with salmon calcitonin (sCT), gastrointestinal absorption of the sCT is markedly enhanced in rodents and monkeys and humans.

### Study design

The study was a randomized, partially-blind, placebo-controlled, single dose, exploratory crossover study to assess the PK and PD of 0.8 mg SMC021 in healthy postmenopausal women when taken in the morning with different volumes of water (50 or 200 ml) and at different times before a meal (10, 30, or 60 minutes). Subjects were blinded to treatment with SMC021 and placebo, but were not blinded to water volume intake and pre-meal dosing time. The PK and PD profiles of Miacalcic^® ^200 IU NS were additionally assessed in an open-label manner in a small subset of subjects.

Females fulfilling the inclusion criteria of being generally healthy ambulatory female volunteers, aged between 40–70 years and having passed a natural or surgically-induced menopause at least 5 years before entering the study and being without diseases or medications known to affect bone metabolism were allowed to participate. The study was of a 5-period, 10-treatment, 56-sequence crossover design. Subjects were randomly allocated to a sequence. The study contained 10 possible treatments, comprising a combination of study medication of SMC021, placebo, and Miacalcic^® ^NS), water volume given with the tablet (50/200 ml water, and dosing time to meal (10, 30 or 60 minutes pre-meal) as outlined in Table 1. To reduce the burden of treatment to the subjects, an incomplete cross-over design was used. All subjects received five of these treatments, according to pre-defined sequences, with a minimum of a 3 day wash-out period between each dose. No sequence included more than one dose of placebo, and no sequence included the same treatment twice. The subjects were blinded to treatment with SMC021 and placebo but not to water volume or pre-meal dosing time. In addition, no blinding was used for Miacalcic^® ^200 IU NS. Placebo treatment was equally distributed across the different meal timings to balance the cross-over design.

**Table 1:** Treatments and Dosing Regimens

Treatment	Number	Study medication	Water volume	Dosing time pre-meal
1	*n *= 40	SMC021 0.8 mg	50 ml	10 min
2	*n *= 40	SMC021 0.8 mg	50 ml	30 min
3	*n *= 40	SMC021 0.8 mg	50 ml	60 min

4	*n *= 40	SMC021 0.8 mg	200 ml	10 min
5	*n *= 40	SMC021 0.8 mg	200 ml	30 min
6	*n *= 40	SMC021 0.8 mg	200 ml	60 min

7	*n *= 10	Placebo	200 ml	10 min
8	*n *= 10	Placebo	200 ml	30 min
9	*n *= 10	Placebo	200 ml	60 min
10	*n *= 10	Miacalcic^®^200 IU NS	N/A	60 min

After an overnight fasting period for at least 10 hours, the subjects received the study medication at 08:00. For subjects receiving either SMC021 or matching placebo, either 50 or 200 ml of water was given with the tablet, depending on the instructions provided for the specific dose in the treatment sequence. For subjects receiving Miacalcic^® ^NS, no water was given with the dose. Except for the fluid given at the time of drug intake and the drink given as part of the standard breakfast, no fluid intake was allowed from 1 hr before until 2 hrs after dosing. Otherwise, subjects had a fluid intake of at least 200 ml every 4 hours, starting from the time of dosing, in addition to fluid taken with meals and medication. Apart from the breakfast meal, no other food was consumed during the time interval of blood sample collections.

For the evaluation of the pharmacokinetics and pharmacodynamics, blood samples were collected immediately prior to dosing (time 0 minutes), and at the intervals of 5, 10, 15, 30, 45 minutes, 1, 1 1/2, 2, 2 1/2, 3, 4, 5, and 6 hours after dosing. Plasma and serum samples were stored at -20°C until analysis.

The concentration of plasma sCT was measured by as chemiluminescence-based assay that previously have been described [7]. Values measured below the lower limit of quantification of 2.5 pg/ml was assigned the value of 2.5 pg/ml. The assay was of the two-site immunometric type, employing two antibodies, one biotinylated and the other acridium labeled. Specificity has been tested against synthetic fragments of sCT and against human as well as eel calcitonin and negligible interaction has been found over the range of standard curve. The lower limit of quantification (LLOQ) was 2.5 pg/mL. The quality control (QC) samples, ranging from 2.5 pg/mL to 700 pg/mL, were prepared daily and measured in 3 to 5 replicates. The overall accuracy and precision (CV) of QC samples, measured on 11 different days was 101.3% and 10.1% for 2.5 pg/mL concentration and 94.3% and 6.0% for 700 pg/mL concentration, respectively.

The Serum CTX-I test is a sandwich enzyme enzyme-linked immunosorbent assay (ELISA) employing two monoclonal antibodies both recognizing the C-telopeptide of the α1-chain in type I collagen [26]. The monoclonal antibodies, i.e. MAb F1103 and MAb F12, recognize the eight amino acid sequence EKAHD-β-GGR, where D-β-G denotes an isomerised bond between aspartate and glycine, and both antibodies require the presence of a free C-terminal arginine for binding. Cathepsin K, secreted by the osteoclast, is responsible for the proteolytic cleavage exposing the free C-terminal arginine [27]. The sandwich construction assures that only cross-linked di-peptides, i.e. EKAHD-β-GGR × EKAHD-β-GGR, are measured by the Serum CTX-I ELISA. The measuring range is 0.020–3.380 ng/ml, and in this range the intra- and interassay coefficient of variation is <3.0 and <10.9%, respectively, and the dilution recovery 103%. The reference range (mean (95% confidence interval)) for postmenopausal and premenopausal women as well as men is 0.439 ng/ml (0.142 – 1.351 ng/ml), 0.287 ng/ml (0.112 – 0.738 ng/ml), and 0.294 ng/ml (0.115 – 0.748 ng/ml), respectively, according to the manufacturer (Immunodiagnostic Systems Nordic, Herlev, Denmark) [26].

The study was conducted in accordance with Helsinki Declaration II, and approved by local Ethical Committees (ClinicalTrails.gov NCT00395395). Written informed consent was obtained from all participants. A copy of the written-consent form is on file, and is available for the editor-in-chief of this journal for review.

### Statistical analysis

The primary objective was to compare the effect on the PK profile of 0.8 mg sCT, as measured by C_max _and AUC_0–4 hrs_, of the amount of water intake (50 ml or 200 ml) and the effect of meal timing after the dosing (10, 30 or 60 minutes), a sample size of 56 subjects was determined to have at least 83% power to reject the null hypothesis that the absolute difference in the loge transformed sCT Cmax value was above 0.693 using a 2-sided *t*-test at the 5% significance level (transformed back to the original scale, the null hypothesis corresponds to a ratio below 0.5 but greater than 2). For the calculation, the mean square error of the loge transformed sCT Cmax was estimated to be 1.0 and the expected ratio between the means was assumed to be 0.95.

The trapezoidal method was applied for calculation of AUC of plasma sCT and of relative change in serum CTX-I after dosing. The relative value of serum CTX-I in postdose samples was calculated as percentage of the individual pre-dose value within each treatment period. The relative change of serum CTX-I was determined as 100% minus the relative value of serum CTX-I. The AUC of plasma sCT, and the time course data of plasma sCT, and serum CTX-I were logarithmically transformed to obtain normality and symmetry of variances. Comparison between treatments of C_max _and AUC of plasma sCT, and AUC of relative change in serum CTX-I was performed using a linear mixed effect model with the variable as the response variable, and treatment (1, 2, 3, 4, 5, 6, and 10) and period (1, 2, 3, 4 and 5) as fixed effects, and subject as a random effect. A difference was considered significant if p-value was less than 5%. No adjustments for multiplicity were performed. The statistical calculations were performed using the SAS software package (release 9.1, SAS Institute Inc., Cary, NC, USA).

## Results

The demographic characteristics of age, height, weight, and BMI of the study participants are given in Table 2. The subjects were aged between 56 and 70 years. All subjects attended all 5 study periods.

**Table 2:** Characteristics of study population (*n *= 56)

	Mean (SD)	Range
Age (Yrs)	64.2 (3.5)	56 – 70
Height (m)	163.9 (5.9)	151 – 177
Weight (kg)	68.4 (7.8)	53.4 – 87.5
BMI (kg/m^2^)	25.5 (2.8)	20.1 – 30.1

**Figure 1:**
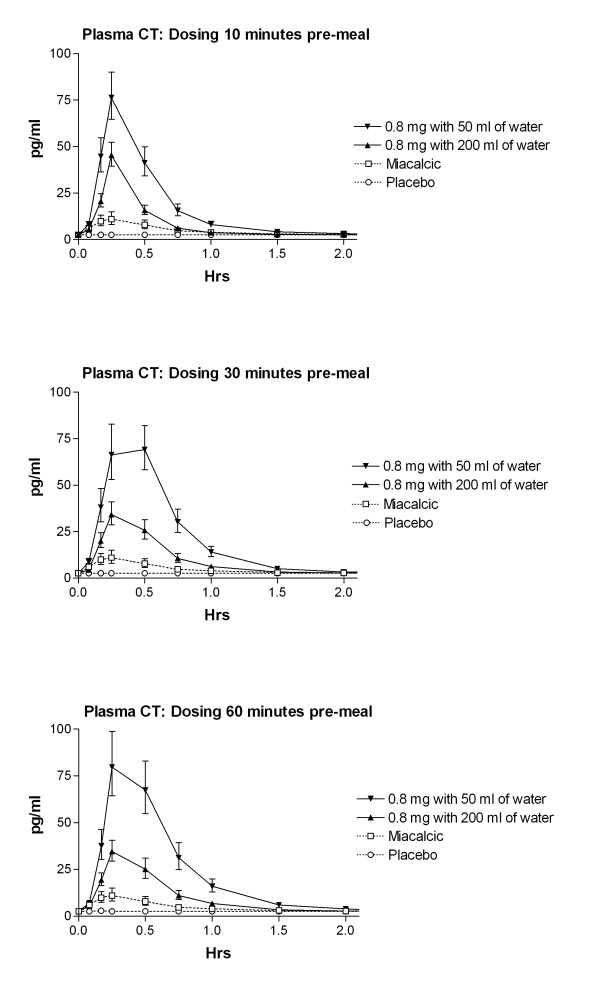
**Time course of plasma sCT 2 hours following one single dose of 0.8 mg with either 50 or 200 ml of water compared of that of Nasal sCT, as indicated in the legend.** Values given are geometric mean ± 1 SEM.

**Figure 2:**
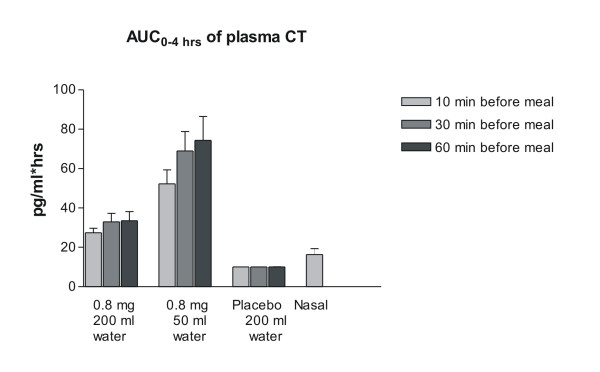
**AUC of plasma sCT in the 2 hours following one single dose of 0.8 mg with either 50 or 200 ml of water compared of that of Nasal sCT, as indicated in the legend.** Values given are geometric mean ± 1 SEM.

The effect of water volume and time between dosing and a subsequent meal on the absorption of sCT is shown in Figure 1. The sCT appeared rapidly in plasma reaching maximum concentrations approximately 15 minutes after drug intake with minimal if any effect of water and meal time on T_max _(Table 3). However, the level of the maximum concentration, C_max_, and the AUC of plasma sCT in the period between dosing and 4 hours post-dose, was dependent on the amount of water intake and timing of meal. A two- to three fold higher absorption was attained with the smaller amount of water of 50 ml as compared with 200 ml (Figure 2) and Table 3, e.g. in the women who fasted for 1 hour subsequent to dosing, the AUC_0–4 hrs _of sCT was 74.4 (pg/ml × hrs) when SMC021 was taken with 50 ml of water and 33.6 (pg/ml × hrs) when taken with 200 ml of water. The effect of water volume was statistically highly significant irrespective of post-meal timing (10, 30 and 60 minutes post-meal time: p < 0.001). The AUC_0–4 hrs _of nasal calcitonin was 16.4 pg/ml × hrs, lower than those observed with administration of 0.8 mg of oral sCT, although the comparison is based on a smaller number of observations There was increased absorption with delayed intake of meal, especially for the groups with intake of 50 ml of water, but the effect of timing of meal was far less pronounced than that of water. The delayed intake of water significantly increased sCT when comparing 10 and 60 minutes, and was borderline significant at 30 minutes compared to that of 10 minutes. Following the absorption phase, sCT was rapidly eliminated from the circulation with an elimination half-life T_1/2 _between 11 and 20 minutes comparable between all groups (data not shown).

**Table 3:** Pharmacokinetic profile

Study medication	Water Volume	Dose pre-meal	AUC_0–4 _pg/ml × hrs	C_max _pg/ml	T_max _minutes
SMC021	50 ml	10 min	52.2 (46.0–59.4)	103 (89–118)	15 (10–23)
SMC021	50 ml	30 min	68.9 (60.2–78.9)	125 (110–144)	15 (15–30)
SMC021	50 ml	60 min	74.4 (64.0–86.5)	145 (125–168)	15 (15–30)

SMC021	200 ml	10 min	27.4 (25.2–29.7)	52 (45–59)	15 (10–15)
SMC021	200 ml	30 min	32.9 (29.1–37.3)	56 (48–67)	15 (15–30)
SMC021	200 ml	60 min	33.6 (29.5–38.1)	49 (42–58)	15 (12.5–30)

Miacalcic	N/A	60 min	16.4 (13.8–19.4)	11.4 (8.3–15.5)	15 (10–15)

AUC_0–4 _and C_max _is given as geometric mean (mean ± 1 sem), and T_max _as median (25–75 percentile)

**Figure 3:**
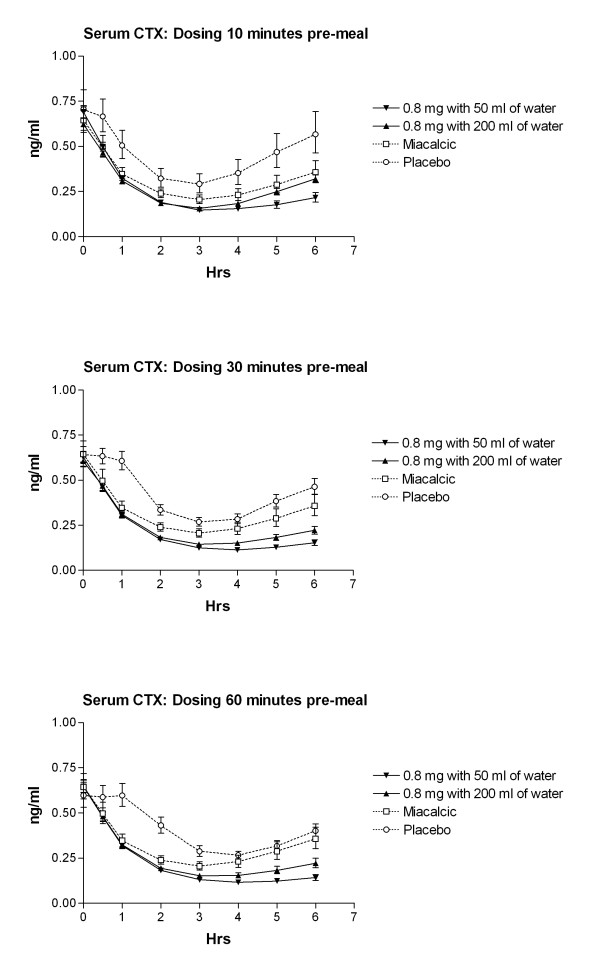
**Time course of absolute level of serum CTX-I in the 6 hours following one single dose of 0.8 mg with either 50 or 200 ml of water compared of that of Nasal sCT, as indicated in the legend.** Values given are geometric mean ± 1 SEM.

**Figure 4:**
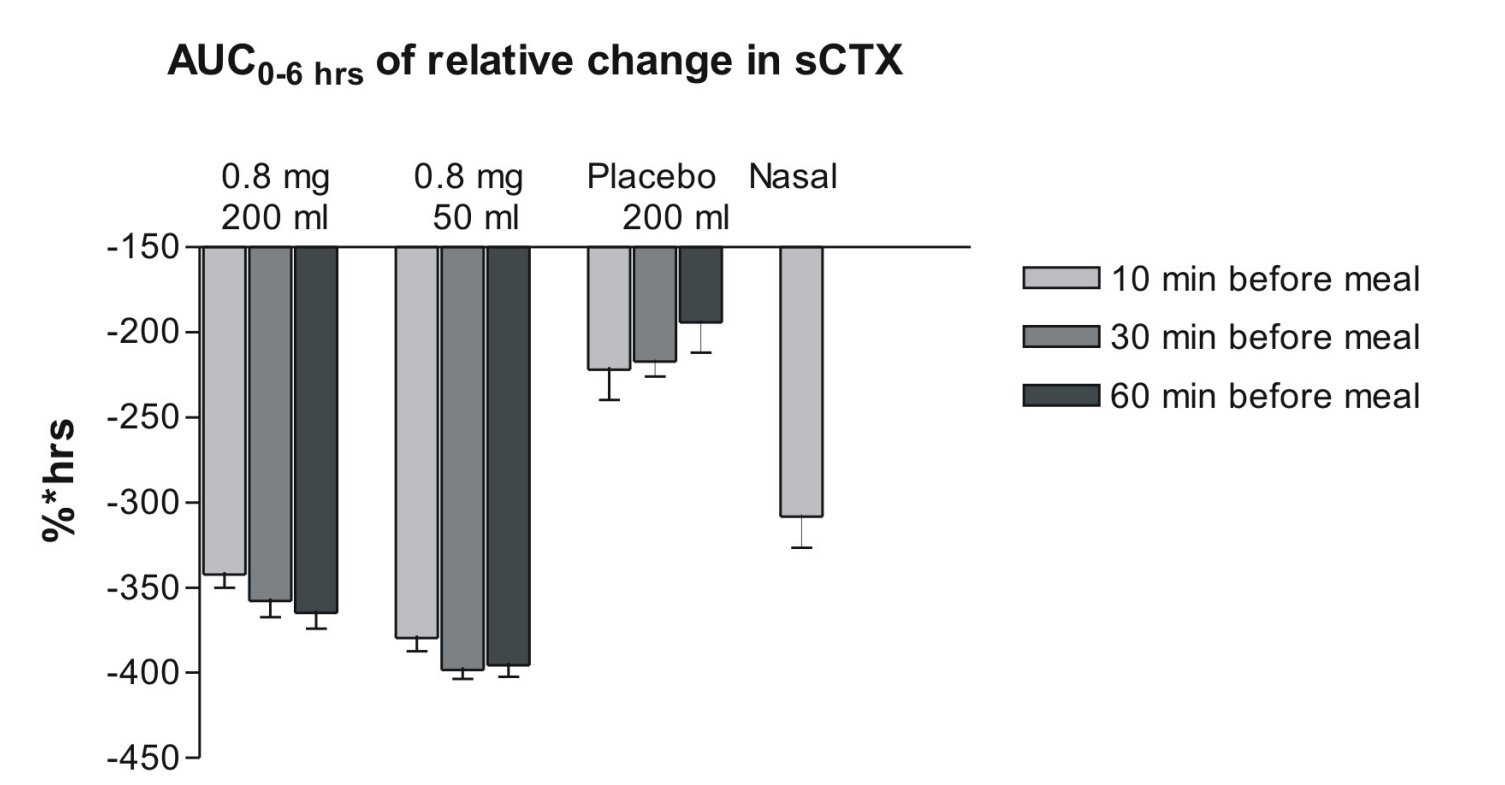
**AUC of relative change in serum CTX-I in the 6 hours following one single dose of 0.8 mg with either 50 or 200 ml of water compared of that of Nasal sCT.** Values given are geometric mean ± 1 SEM.

In accordance with the absorption of sCT, the water volume and time of meal post-dosing revealed a similar pharmacodynamic effect on bone resorption, measured by concentration of serum CTX-I over the 6 hours post-dose (Figure 3 and 4). Overall, dosing with SMC021 resulted in a significant suppression of serum CTX virtually reaching the same level of nadir 3 to 4 hours post-dose irrespective of water volume and time of meal post-dosing (Figure 3). However, the remission to baseline started earlier in subjects who took the medication with 200 ml of water as compared to that with 50 ml. In similar context, return to baseline values occurs earlier in subjects who had intake of meal 10 minutes after dosing as compared to those having meal 30 and 60 minutes after dosing. Taken together, and as presented in Figure 4, the largest treatment effect measured as the AUC of change in relative values of serum CTX-I during the 6 hours post-dose was achieved for the oral dose of 0.8 mg taken with 50 ml of water, 30 or 60 minutes before meal intake. The placebo-adjusted treatment effect of the dose given 60 minutes prior to meal was -171 (% × hrs) and -202 (% × hrs) for 0.8 mg SMC021 taken with 200 ml and 50 ml of water, respectively, and -114 (% × hrs) in the subjects receiving nasal calcitonin.

## Discussion

Oral drug delivery of small peptides is hampered by an array of obstacles that are best illustrated by the complete lack of peptides in oral formulations approved by the FDA. We have investigated the effect of water and meal time on the absorption of a single tablet of 0.8 mg of salmon calcitonin given orally by pharmacokinetic and pharmacodynamic parameters and compared them to that of the nasal spray of calcitonin given in a dose of 200 IU. These data are the first to demonstrate oral delivery of a small peptide in which optimization of drug intake parameters including amount of water intake can augment bioavailability and subsequently improve PD effect over 6 hours. The currently presented results are especially important in determining the optimal oral delivery of calcitonin, and they may be of use in development of other peptides to be formulated for oral administration.

An array of attempts have been made to formulate calcitonin for oral administration [9,10,12-16,19,20,23,25,28]. 5-CNAC is a novel compound, that already has been proven to be useful clinically [6,7]. Even in the face of already proven clinical pharmacodynamic effects [6,7], administration of this novel formulation may possibly be optimized further to enhance uptake and thereby improve clinical effect.

The current study clearly suggests that drug uptake of SMC021 is influenced by the amount of water given with the tablet. A water volume of 50 ml resulted in a two- to three-fold higher absorption of sCT in comparison with a volume of 200 ml of water. This doubling of absorption was seen irrespective of the timing of meal after dosing, i.e. meal given 10, 30, and 60 minutes post dosing, suggesting an impact of water intake on digestion and absorption. Secondarily, this resulted in improved efficacy, measured by the biochemical marker of bone resorption, serum CTX-I. These data are the first to demonstrate that water intake has an important effect on oral peptide uptake with the Eligen technology, improving bioavailability as much as 400%, and even more if placebo corrected. This finding may be extended to other formulations.

Previous studies have indicated that the bioavailability of some drug substances is heavily influenced by meal time [29-31] compared to that of fasting state [32-34]. The effect of meal timing on the oral delivery of small proteins/peptides has been investigated less than that of small molecules. The present study investigated the effect of two water volumes (50 mL and 200 mL) and meal intake (10, 30, and 60 minutes) after drug dosing to determine optimal dosing conditions. Dosing 60 minutes prior to meal time with 50 ml of water was significantly better compared to 10 minutes. This was borderline significant for 30 minutes. The results of the study are in alignment with previous studies, documenting the strong suppression of drug uptake when food is taken concomitantly [35,36]. However these interactions are highly drug-specific, which hampers the general conclusions drawn, as lipophilic, hydrophilic and proteins all have different characteristics.

Biochemical markers of bone resorption such as serum CTX-I have on previous occasions been demonstrated to be independent predictors of fracture risk and predictive of drug efficacy [27,37]. Serum CTX-I, which is an osteoclast specific generated collagen type I fragment [38], was significantly inhibited by oral sCT treatment to a greater extent than by the nasal formulation over 6 hours. This may be interpreted as a potential better efficacy. Thus, these data suggest the possibility of efficacy superior to that of the nasal spray observed in previously clinical trials [39].

Bone resorption exhibits marked diurnal variation [35,40], most of which is linked to a post-prandial decrease [27,36]. In the current study, this effect was clearly observed after the placebo treatment when the decrease in CTX-I started at meal time.

## Conclusion

In conclusion, these data suggest that SMC021 given with 50 ml of water prior to meal time results in greater sCT bioavailability than when given with 200 ml of water, and that timing the dose 60 minutes pre-meal results in greater sCT bioavailability than 10 minutes pre-meal. Also, the sCT bioavailability and CTX-I pharmacodynamic effect appear to be greater compared to that of nasal calcitonin. Further clinical studies are needed to evaluate the safety and efficacy of SMC021 for the treatment of osteoporosis.

## Abbreviations

CT: Calcitonin; PK: Pharmacokinetic; PD: Pharmacodynamic; sCT: Salmon calcitonin; CTX-I: C-termial collagen type I degradation; LLOQ: Lower limit of quantification; QC: Quality control; AUC: Area under the curve; ELISA: Enzyme enzyme-linked immunosorbent assay.

## Competing interests

Morten Karsdal, Bente Jul Riis and Inger Byrjalsen are full time employee of Nordic Bioscience, a company involved in the development of oral calcitonin. MK has revived consulting fees from Novartis. Claus Christiansen is the CEO of Nordic Bioscience.

## Authors' contributions

CC and BJR designed the study and reviewed the last version of the manuscript. IB performed statistical analysis and participated in drafting of the manuscript. MAK analyzed the data, drafted the first manuscript and finalized the last version of the manuscript. All authors approved the final version of the manuscript.

## Pre-publication history

The pre-publication history for this paper can be accessed here:


